# Mr. Bartley, on Digitalis

**Published:** 1801-03

**Authors:** O. W. Bartley

**Affiliations:** Surgeon. Bradford, Wilts


### *To the Editors of the Medical and Phyfical Journal.*

"Segnius irritant animos demiffa per aures

Quam quæ funt oculis fubjecta fidelibus." HOR.

GENTLEMEN,

AFTER the many learned difquifitions and numerous inftances already advanced by eminent profeffors in favour of the Digitalis Purpurea, the obfervations I am about to adduce may appear trifling and infignificant; but I conceive it behoves every practitioner to contribute his information, however fmall, to the general fund of knowledge, efpecially if it may tend to the decifion of a queftion fo important as the efficacy or inefficacy of a remedy, in a difeafe by whofe ravages thoufands of our natives are annually facrificed. A difeafe which, in our variable climate, deftroys the bloffoms of our youth, precipitates into an untimely grave the darling hopes of many worthy families, and perpetually adds to the number of the widows and fatherlefs.—I have at prefent an opportunity of ftating a fingle inftance only of its effects in phthifis pulmonalis, which I hope will not be rejected on account of its being folitary, as I do not communi-

 cate cate it with a view of prejudicing the public in favour of a new remedy; but, unactuated by a love of novelty, I would recommend it to serious examination and attention, that every practitioner may, on the evidence of his own experience, reject or approve the use of the Fox-glove in a disease demanding such earnest consideration as that of pulmonary consumption.

I shall proceed to a statement of my case, which I shall endeavour to transcribe with impartiality, and as much accuracy as I am capable of; being pre-determined, on my first exhibition of the Digitalis, to make known its effects, whether its administration was attended with success or otherwise.

In November last I was required to attend on Thomas Lane, a cloth-worker, residing in this town, aged 27 years; he was pre-disposed to phthisis both from *forma corporis* and temperament. I found him in the purulent stage of that disorder. He had hectic fever with marked exacerbations; expectoration copious and *evidently purulent*, profuse nightly perspirations, acute pain in the side, incessant cough, disturbed sleep, and considerable dyspnoea, accompanying great emaciation and debility. The bowels, tongue, and skin were natural, the appetite very much impaired, and pulse 120. I learned from his account that some months before, a watry eruption (I suppose erysipelatous) attended with great heat and inflammation, appeared in many parts of his body, but was more particularly confined to his face, neck, and breast. Whilst in this condition he was imprudent enough to assist in drawing a fish-pond, and immediately caused a retropulsion of the eruption, from which time he dates the commencement of the symptoms before enumerated.—Nov. 29, Began the use of the saturated tincture; pulse 119. ℞. Tinct. Fol. Digital. gt. xv. ex cyatho lactis ter in die.—30th, Had slept rather better, the cough was less troublesome, and the expectoration somewhat diminished, night sweat rather less profuse, appetite bad, bowels, tongue and skin natural, pulse 104. In the morning an eruption resembling nettle-rash appeared over the whole surface of the body, which entirely receded before noon.—Dec. 1, A bad night, watchfulness in the early part being occasioned by a griping pain in the bowels, with diarrhoea, which terminated towards morning, when he enjoyed undisturbed repose for some hours; night sweat trifling. In the morning an erysipelatous eruption appeared in every part of the body; skin hot and dry. The tongue a little furred, very little pain in the side, cough and expectoration (to use his own expression) not half so frequent. In the forenoon he complained of slight nausea and vertigo; the appetite bad; pulse 94.—Cont. Dos. As he complained of much thirst, I ordered him a cooling beverage with Tart. Crystall. and Fruct. Tamarind.—2d, Slept better, better, vomited towards morning, no night ſweat, bowels ſoluble, tongue furred, ſkin hot and dry; the eruption has partly diſappeared. Complains of ſlight pain in the hypochondriac and hypogaſtric regions; trifling vertigo; cough and expectoration the ſame as on the preceding day; appetite bad; pulſe 73. Ordered him to omit one doſe of his mixture.—3d, Slept conſiderably better; does not complain of nauſea or vertigo; no nightly perſpiration; bowels laxative, tongue and ſkin healthy. The eruption has entirely diſappeared; pain in the ſide trifling; cough more frequent, and expectoration increaſed; appetite much better; pulſe 85.—Aug. Dos. ad gt. xx.—4th, Had neglected one doſe of the tincture; ſlept well, trifling ſweat, no nauſea or vertigo, bowels ſoluble, tongue healthy; a ſlight appearance of the eruption; pain in the ſide continues; cough more frequent, and expectoration more copious; appetite tolerable, pulſe 80. Cont. Dos.—5th, Sleep interrupted by the frequency of the cough, no nauſea or vertigo, no colliquative perſpiration, b. t. and ſkin natural; the expectoration aſſumes a frothy mucous appearance with little pus. The pain in the ſide is leſs; appetite good; is much improved in ſtrength, and his countenance wears a more healthy aſpect; pulſe 90. Cont. Dos.—6th, Slept little better; cough and expectoration increaſed; pain in the ſide troubleſome; b. t. and ſkin natural; pulſe 75. Cont. Dos.—7th, Sleep almoſt prevented by frequency of cough; expectoration more copious; trifling night ſweat; pain in the ſide more acute; b. t. and ſkin natural; appetite tolerable; pulſe 78. Cont.—8th, Had tranſgreſſed the regimen I had preſcribed for him by drinking freely of ſtrong malt liquor; in conſequence he had ſlept ill; experienced a copious return of the night ſweat; cough, expectoration, and pain in the ſide conſiderably increaſed; bowels, tongue, and ſkin natural; appetite bad; pulſe 100. Aug. Dos. ad gt. xxv.—9th, Was prevented by other engagements from ſeeing him.—10th, Complains of conſiderable debility; a propenſity to ſleep when not diſturbed by the cough, which is very troubleſome; expectoration more profuſe, but not more purulent; pain in the ſide very acute; appetite very bad; colliquative night ſweat continues; b. and ſk. natural; tongue foul; pulſe 88. Cont.—11th, Sleep ſtill prevented by increaſe of cough and expectoration; night ſweats ſtill copious; pain in the ſide continues troubleſome; feels no inclination for food, and labours under very conſiderable debility; b. t. and ſk. natural; pulſe 75. Cont.—12th, Viſited him early in the morning; ſleep had forſaken him during the whole of the night; the increaſe of cough is conſiderable, attended with violent vomiting; extreme debility and acute pain in the ſide; and his ſtomach rejects jects food; the expectoration is copious, but with little appearance of pus, which circumſtance alone induced me to continue the Digitalis, the other unfavourable ſymptoms almoſt inclining me to deſiſt from its further uſe; pulſe 50.—Ordered him to omit taking the drops until bed-time.—13th, The urgent ſymptoms appear much more favourable; he had ſlept better; colliquative perſpiration diminiſhed; appetite amended; cough and expectoration leſs; b. t. and ſk. natural; pulſe 73. Cont. —14th, Slept much better; no night ſweat; cough leſs frequent; expectoration much diminiſhed; appetite improved; pain in the ſide ſomewhat relieved; b. t. and ſk. natural; pulſe 74. Cont.—15th, Sleep much diſturbed; cough and expectoration again increaſed; as alſo the night ſweat; pain in the ſide rather ſevere; appetite bad; b. t. and ſk. natural; pulſe 80. Cont.—16th, Every ſymptom remained much the ſame as on the day preceding; appetite rather better; b. t. and ſk. natural; pulſe 75. Cont.—17th, Slept tolerably well, very little perſpiration; cough and expectoration leſs; appetite better; b. t. and ſk. natural; pulſe 73. Cont.—18th, Very bad night; complains of intolerable head-ach; anxiety and languor extreme; no appetite; b. t. and ſk. natural; Pulſe 63.—19th, Watchfulneſs, anxiety, head-ach, &c. increaſed; cough much leſs frequent, and expectoration much diminiſhed; no night ſweat; appetite very bad; b. t. and ſk. natural; pulſe 59.— On inquiry, I found the attendant had deviated from the directions I had given her, by adminiſtering gt. xxx. pro gt. xxv. of the tincture each doſe; Cont. gt. xxv.—20th, The ſymptoms were rather relieved; had ſlept tolerably well; cough and expectoration continue to decreaſe; appetite better; b. t. and ſk. natural; pulſe 65. Cont.—21ſt, Slept much better; experiences little inconvenience from the cough; the expectoration very inconſiderable, and *not at all purulent*; a ſlight return of the colliquative perſpiration towards morning; has ſtill ſlight head-ach; appetite improved; b. t. and ſk. natural; pulſe 70. Cont.—22d, Every ſymptom appears more favourable; b. t. and ſk. natural; pulſe 68. Cont.—23d, The unfavourable ſymptoms continue rapidly to ſubſide; ſleeps well; appetite rather craving; b. t. and ſk. natural; pulſe 73. Cont.—24th, Sleep found and refreſhing; no colliquative ſweats; the appetite very good; cough ſeldom occurs, and the expectoration is trifling; no pain in the ſide; b. t. and ſk. natural; pulſe 70. Cont. 25th, Every ſymptom ſtill more favourable; pulſe 73. —26th, Still continues to amend; pulſe 70. Cont.—27th, No cauſe for diminution of hope, as favourable circumſtances increaſed; pulſe 71.—28th, The diſeaſe continues to give place to returning health; pulſe 71. Cont.—29th, Every thing ſeems to to indicate a fpeedy though progreffive recovery; pulfe 70. Cont.—30th, Complains of watchfulnefs occafioned by acute pains in the knee-joints, apparently rheumatic; cough and expectoration very inconfiderable; pulfe 69.—℞. Pulv. Ipecac. comp. ℈j. hora fomni fumend. ℞. Linim. fapon. tinct. opii. aa. ℥j. M. F. Linim. part. dolent. fæpe applicand. Cont. tinct. digital.—31ft, The rheumatic affection has yielded to the fudorific powder and anodyne liniment. He fcarcely ever experiences any occurrence of cough, except a little on firft lying down in his bed; pulfe 73. In fhort, not to tire your patience further by ufelefs repetitions, I fhall briefly obferve, that he foon became convalefcent; nearly a month has elapfed fince he has experienced the fmalleft inconvenience from cough or any other unfavourable occurrence. He at prefent purfues his general occupation with his ufual alacrity.

With the moft fincere wifhes for the long continuance and profperity of your very excellent and laudable publication, I remain, &c.

